# Pediatric COVID-19: Report From Indonesian Pediatric Society Data Registry

**DOI:** 10.3389/fped.2021.716898

**Published:** 2021-09-23

**Authors:** Antonius H. Pudjiadi, Nina Dwi Putri, Hikari Ambara Sjakti, Piprim Basarah Yanuarso, Hartono Gunardi, Rosalina Dewi Roeslani, Ade Djanwardi Pasaribu, Lies Dewi Nurmalia, Catharine Mayung Sambo, I Dewa Gede Ugrasena, Santoso Soeroso, Armijn Firman, Heru Muryawan, Darmawan Budi Setyanto, Endah Citraresmi, Jaya Ariheriyanto Effendi, Lathiefatul Habibah, Prillye Deasy Octaviantie, Indriyanti Natasya Ayu Utami, Yogi Prawira, Nastiti Kaswandani, Anggraini Alam, Kurniawan Taufiq Kadafi, Aman B. Pulungan

**Affiliations:** ^1^The Indonesian Pediatric Society, Jakarta, Indonesia; ^2^Department of Pediatrics, Faculty of Medicine, Cipto Mangunkusumo National Central Hospital, Universitas Indonesia, Jakarta, Indonesia; ^3^Department of Pediatrics, Faculty of Medicine, Dr. Soetomo Hospital, Universitas Airlangga, Surabaya, Indonesia; ^4^Department of Pediatrics, Faculty of Medicine, Hasan Sadikin Hospital, Universitas Padjajaran, Bandung, Indonesia; ^5^Department of Pediatrics, Faculty of Medicine, Dr. Kariadi Hospital, Universitas Diponegoro, Semarang, Indonesia; ^6^Department of Pediatrics, Harapan Kita Women and Children Hospital, Jakarta, Indonesia; ^7^Department of Pediatrics, Fatmawati Hospital, Jakarta, Indonesia; ^8^Department of Pediatrics, Faculty of Medicine, Dr. Saiful Anwar Hospital, Universitas Brawijaya, Malang, Indonesia

**Keywords:** COVID-19, Indonesia, children, mortality, comorbidities

## Abstract

**Background:** Indonesia has a high number of COVID-19 cases and mortalities relative to not only among the Asia Pacific region but the world. Children were thought to be less affected by the virus compared to adults. Most of the public data reported combined data between adults and children. The Indonesian Pediatric Society (IPS) was involved in the COVID-19 response, especially in the area of child health. One of IPS's activities is collecting data registries from each of their chapters to provide a better understanding of COVID-19 in children.

**Objective:** The objective of this study was to share the data of suspected and confirmed COVID-19 cases in children from IPS's COVID-19 data registry.

**Method:** This is a retrospective study from the IPS's COVID-19 registry data. We collected the data of COVID-19 in children during March to December 2020 from each of the IPS chapters. We analyzed the prevalence, case fatality rate (CFR), age groups, diagnosis, and comorbidities of the children diagnosed with COVID-19.

**Result:** As of December 21, 2020, there were 35,506 suspected cases of children with COVID-19. In total, there were 522 deaths, with a case fatality ratio (CFR) of 1.4. There were 37,706 confirmed cases with 175 fatalities (CFR 0.46). The highest mortality in confirmed COVID-19 cases was from children ages 10–18 years (42 out of 159 cases: 26%). The most common comorbidity and diagnosis found were malignancy (17.3%) and respiratory failure (54.5%).

**Conclusion:** The CFR of confirmed COVID-19 cases in children in Indonesia is high and should be a major public concern.

## Introduction

Indonesia is situated in the world's largest archipelago, comprising more than 17,000 islands with 34 provinces. These geographic characteristics present various public health challenges and lead to inequalities in public health services. In late December of 2019, a mysterious infectious respiratory disease emerged in China (1). On February 11, 2020, WHO declared the disease COVID-19, caused by SARS-CoV-2 (2). They subsequently declared COVID-19 a global pandemic on March 11, 2020. Meanwhile, in Indonesia, the first case was recorded on March 2, 2020. Ten months into the pandemic in Indonesia, the number of cases had grown to a total of 671,778, with the number of total deaths 20,085 (as of December 21, 2020) (3). This number has put Indonesia in the top three Southeast Asian countries with the highest number of COVID-19 cases and ranked in the top 20 worldwide (4). Most publications relating to COVID-19 have been focused on adults, despite the fact that Indonesia is reportedly one of the countries with the highest COVID-19 death rate among children (5). The aim of this paper is to provide a big picture data of children with confirmed COVID-19 in Indonesia based on IPS data.

## Methods

Independent pediatricians collected and reported cases of COVID-19 on online spreadsheet created by the IPS COVID-19 task force. The data was collected from IPS chapters COVID-19 weekly meeting and represented confirmed COVID-19 cases to support the Ministry of Health. This provision of COVID-19 pediatric data is crucial especially during the early pandemic as the official dashboard of the Ministry of Health (MoH) for the pediatric population was still in progress. The data collection for this study was conducted from March to December 2020 following the first identified COVID-19 case in Indonesia. The study adheres to the Declaration of Helsinki and its amendments. Neither identity nor personal data of patients were collected for this study. This study was reviewed and approved by the Ethical Committee of Faculty of Medicine Universitas Indonesia (S-72/UN2.F1/ETIK/PPM.00.02/2021). Data collected by total sampling include subjects' provincial origin, age distribution, comorbidities, mortality outcome, and cause of death. For each mortality outcome, any comorbidities were recorded. This data did not represent the whole country's data as it reports individual pediatricians case findings. For a more comprehensive picture, we also report the data published by the Ministry of Health (MOH) as a comparison. These data by MOH Health account for nationwide RT-PCR-confirmed cases as reported by a certified laboratory to the MOH and were extracted from the covid19.go.id website (6). The website was supervised by the Indonesian COVID-19 Task Force which is the government official page for COVID-19 information. The case definition follows the clinical criteria of COVID-19 published by the World Health Organization (WHO). Data were presented as descriptive data under frequency and questionnaire, generated using Microsoft Excel.

## Results

**Figure 1:**
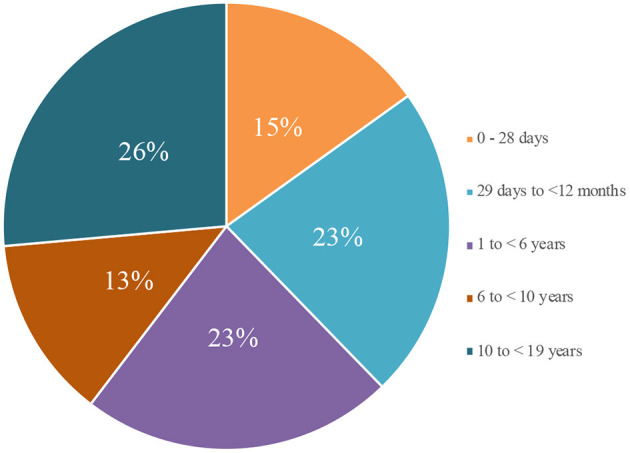
Age distribution among pediatric confirmed COVID-19 cases mortality.

We have collected a total of 72,762 data among our cohort until December 21, 2020. There were 37,706 reported confirmed COVID-19 cases in which 175 cases resulted in death (CFR 0.46). The highest mortality among confirmed COVID-19 cases was from children aged 10–18 years (42 of 159 cases; 26%), followed by children ages 29 days to 11 months 29 days (36 cases; 23%), ages 1–5 years 11 months 29 days (36 cases (23%), ages 0–28 days (24 cases; 15%), and ages 6–9 years 11 months 29 days (21 cases; 13%) (Figure 1).

**Figure 2:**
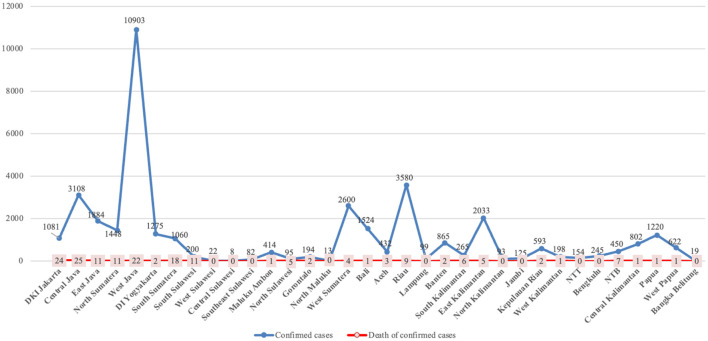
Distribution of confirmed COVID-19 cases according to region of origin.

The regional distribution of COVID-19 cases was recorded. The highest number of confirmed cases was in West Java (10,903), followed by Riau (3,580), Central Java (3,108), West Sumatera (2,600), East Kalimantan (2,033), East Java (1,884), Bali (1,524), North Sumatera (1,448), DI Yogyakarta (1,275), and Papua (1,220). Although Jakarta (13.7%) and South Sumatra (10.3%) were not included in the 10 regions with the highest number of confirmed cases of COVID-19 in children in Indonesia, these regions provided a significant number of child mortalities due to COVID-19 (Figure 2).

**Figure 3:**
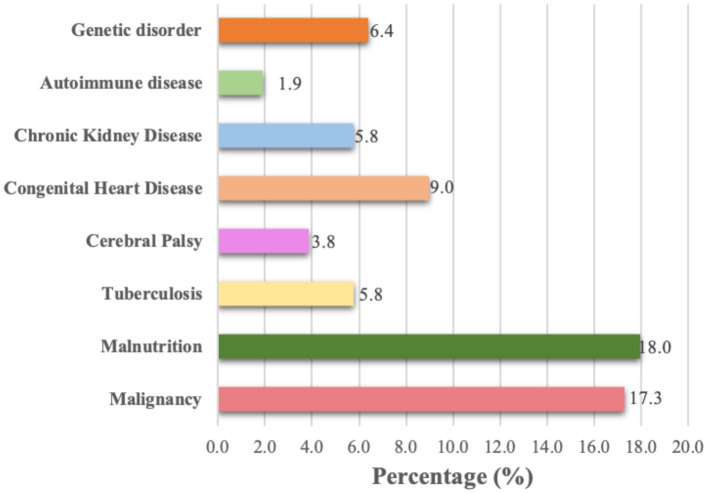
Comorbidities among confirmed COVID-19 non-survivors.

**Figure 4:**
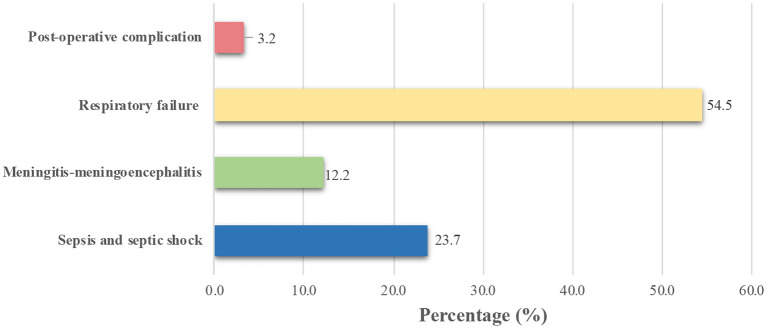
Cause of death among confirmed COVID-19 non-survivors.

Comorbidities and cause of death were recorded with any cases which resulted in mortality. Most of the children who died with COVID-19 had malignancies (17.3%), followed by malnutrition (18.0%) and congenital heart disease (9.0%) (Figure 3). There were 62 confirmed COVID-19 children who died without any comorbidities. In this report, one patient can have more than one comorbidity. The two most frequent diagnoses reported in COVID-19 cases were respiratory failure (54.5%) and sepsis and septic shock (23.7%) (Figure 4).

## Discussion

Amid the COVID-19 pandemic, Indonesia is downgraded as a low-middle-income country (LMIC) by the World Bank. Data of COVID-19 in children from LMICs are not presented well-compared to adult data and data from high-income countries. However, the pandemic is a global health issue that needs cooperation from all the countries worldwide. Transparency of data is very important in order to prevent underestimation of the disease. Indonesia is also significantly affected by the pandemic. It causes delay in achieving SDG targets and increases the pediatric mortality in Indonesia which in turn reduces the overall wellbeing status of Indonesian children.

Based on data from the Ministry of Health (MOH) as of December 21, 2020, children contributed for 77,254 of total 671,778 confirmed cases (11.5%). The proportion of deaths among children aged 0–5 years was 0.9%; aged 6–18 years, 1.8% (total cases 542; total CFR 0.7) (3). From these data, we were able to identify a large discrepancy between the data gathered by the Ministry of Health and the data from our study. This difference might be due to the scope of the data gathered by the IPS being limited to children with confirmed COVID-19 treated by pediatricians (3). Data from the MOH were more likely to have also been collected from asymptomatic children diagnosed from contact tracing positive cases among the adults.

Moreover, the healthcare system in Indonesia is still unevenly distributed due to our large geographical location. According to the data by MOH in 2019, Jakarta, as the capital of Indonesia, had a ratio of 7.16 primary health centers per subdistrict while Bali had 2.11. In contrast, West Papua, one of the more underdeveloped regions in Indonesia, only had 0.28 (7). These numbers illustrate the accessibility for the citizens to obtain health service. The disparity of health service will greatly affect not only the care for COVID-19 patients but also the contact tracing in the first place. This is especially true during the first wave of COVID-19 in Indonesia. Many health facilities suffered from limited resources, including the personal protective equipment for COVID-19 (8).

The PCR testing capability in Indonesia was very low (240 tests per 1 million population) compared to other ASEAN countries such as Philippines (660), Malaysia (3,515), Singapore (16,203), Thailand (2,043), Vietnam (2,119), and Brunei (27,770) (9, 10). The price was also considerably high, with the average of ~900,000 rupiah or USD 60 per test (11). During the earlier period of the COVID-19 pandemic, children were not a priority group for testing. We know that the important variable in determining the prevalence of an infectious disease is the testing capacity. A low testing capacity will lead to delays in diagnosis and treatment. These raised a very important issue, which was case finding of COVID-19, especially those with asymptomatic or mild cases which need no hospitalization. Also, the results for PCR vary among regions, where those from the more rural areas might need several days to weeks for positive results. Suspected cases were not reported in our study, which further increases the CFR of confirmed cases. These hidden cases expose other individuals of contracting COVID-19.

As of October 11, 2020, the Philippines, which has the highest prevalence of COVID-19 in Southeast Asia, has recorded 28,979 children ages 0–19 years with confirmed COVID-19 infection. This means that children only accounted for 0.09% of the population with confirmed COVID-19 in the Philippines. One hundred and thirty-four children had fatal outcomes, resulting in a CFR of 0.46% for children with COVID-19 in the Philippines (10). Other countries in the Southeast Asia region (outside Indonesia and Philippines) did not publish the prevalence of COVID-19 based on age groups on their official websites, so no other comparisons can be made.

In this study, the proportion of COVID-19-related deaths was shown to be higher than the case fatality rate of COVID-19 in the USA and Europe, where the reported mortality rates were 0–0.19 and 0.69% respectively (1, 4, 12). Aside from the low testing capability which results in the low number of positive cases (a smaller denominator would result in higher CFR), several other factors were identified. A previous study in the national referral hospital in Indonesia found that the CFR of COVID-19 in children was as high as 40% (13). This study described that children who came to the hospital were mostly those with severe to critical clinical conditions. All non-survivors had symptoms upon hospital admission, causing a high mortality rate in that study. Similarly, cases with which the children did not visit pediatricians, including those with no symptoms or comorbidities, were not recorded in our study, explaining the higher CFR in the recorded case. Treatment of COVID-19 in Indonesia was not optimal because there was a burden of overload on hospital and medical professionals (14). Finally, most of the provinces were not open for collecting COVID-19 data related to children. It is presumably due to a lack of awareness of COVID-19 cases in children. We also did not have a uniformed reporting of COVID-19 cases in MOH-unregistered laboratories, resulting in a high number of unreported cases.

As the number of deaths reported in pediatric COVID-19 cases was generally low, there is no exact age pattern observable at this current time (15, 16). However, there are several hypotheses that may explain the high prevalence of COVID-19 in babies under 1 year of age as with the result of our study. These theories include high or low angiotensin-converting enzyme 2 (ACE-2) receptors, frequent exposures to viruses from hospitals or medical professionals, and lack of use of personal protective equipment (PPE) such as masks, in children under 1 year of age (17). Newborns from positive mothers are also isolated and treated as suspected cases until negative results are received, indicating a higher number of contact tracing among babies.

In terms of comorbidities, our results are similar to one study looking into pediatric COVID-19 cases admitted to the intensive care unit (ICU). This study was conducted across 46 PICUs in North America in April to March 2020 and found that 86% of patients had at least one comorbidity, which is similar to the findings of our study. It reported that the five most prevalent comorbidities are present in children with medically complex conditions, such as those with long-term dependence on technological support (including tracheostomy) associated with developmental delay and/or genetic anomalies (40%), immune suppression or malignancy (23%), obesity (15%), diabetes (8%), and seizures (6%) (18). A systematic review regarding pediatric COVID-19 patients stated that 79% of pediatric patients were reported to have no comorbidities, and of the 21% with comorbidities, the most common were asthma, immunosuppression, and cardiovascular disease (19).

In our study, we found that malnutrition and malignancy were two of the most common comorbidities among the study participants. The difference in comorbidities in Indonesia with other studies can be explained by the high prevalence of malnutrition in our country (20). Malnutrition exposed children to a higher risk of infections due to reduced immunity compared to healthy individuals. The focus on reducing malnutrition rates in childhood became one of the significant consequences of COVID-19 and impacted several aspects related to access to nutritious foods (21). Based on the data from SSGBI and Susenas, in March 2019 the prevalence of stunting in Indonesia was 27.67%. There were a total of 17 out of 34 provinces in Indonesia where the prevalence of stunting was below the average national rate. Other data showed that four provinces had a prevalence below the WHO standard. These were Bangka Belitung (19.93%), Kepulauan Riau (16.82%), DKI Jakarta (19.96%), and Bali (14.42%) (22). In 2020, the MOH conducted a study to monitor the growth and determinants of nutritional status during the pandemic. This study also showed that the percentage of toddlers that accessed health service for treatment was 52.4%, while 47.6% decided to avoid health facilities (23). Patients with malignancy are also more exposed to COVID-19 as they need to visit highly infectious places including hospitals. A multinational, multicenter study of children and adolescents in Europe found that 25% of their study population had preexisting medical conditions with chronic pulmonary disease and malignancy as the two most common comorbidities (1). The diagnosis in our confirmed cases is similar to other studies where pulmonary, neurological, and multiorgan dysfunctions were found (23, 24).

Even though the IPS study showed the comorbidities and mortality related to COVID-19 in children, it should be noted that the causes of high infant mortality in Indonesia were disturbances in the neonatal period (49.8%), congenital and genetic anomalies (14.2%), pneumonia (9.2%), diarrhea, other gastrointestinal infections (7%), viral hemorrhagic fever (2.2%), meningitis (2%), undernutrition, and metabolic disorders (1.3%) (25). Our efforts in reducing these causes of deaths in children are still ongoing. However, the pandemic made a significant setback in reducing these issues as health resources are still adapting to the more demanding COVID-19 cases. Two most common causes of death in this study were respiratory failure and sepsis/septic shock. Pre-hospital and in-hospital resuscitations became an important key factor in preventing the development of these conditions. This might be tricky during the pandemic era where limited resources were available for the large demand for hospitalization among the positive COVID-19 cases, including the adult cases (14).

Furthermore, the coverage of immunization in children is another topic that must be faced during this pandemic era. Indonesia has not reached the expected level in some regions in Indonesia. Based on health research in 2018, the proportion of complete basic immunization in children was only 57.9% while the target of a strategic plan for 2019 was 93% (25). In addition, a study during the pandemic in 2020 showed that from 250 districts in Indonesia, 81.9% of children (0–23 months) visited health facilities for immunization (26, 27). These recent data showed that parents are still aware of their children's immunization, even during the COVID-19 pandemic. During the first social distancing, the IPS had released recommendation where immunization may be delayed for up to 1 month. This might be in effort to limit the number of visits to primary healthcare facilities to prevent crowding of children. However, to prevent the reemerging of vaccine-prevented disease, IPS could not recommend prolonged delay for immunization even during the pandemic era (28). Although as of December 2020, the immunization for COVID-19 is still being under vigorous research, additional data regarding the correlation between immunization coverage for other diseases and COVID-19 infection are still needed.

This is the first big data of pediatric COVID-19 cases in Indonesia. The main limitation of this study is that we could not provide comprehensive data of the suspected cases. Thus, we are unable to perform statistical analysis to measure the predictors of death in our study population. The study design also did not allow the evaluation of risk or identify modifying variables for COVID-19 mortality. In addition, with the high number of pediatric COVID-19 cases, we predict that the number of multisystem inflammatory syndromes in children (MISC) should be higher. Previous studies in other countries such as the Latin America and UK found a prevalence of 23.2% out of 409 children and 11% in 651 children recruited. However, the authors addressed that this number could be an overestimation of the real cases due to the broad definition of MISC with no confirmatory objective parameters. Further studies are needed in order to identify the exact ratio of MISC among our population. This will be beneficial for early detection and treatment to reduce the morbidity and mortality of Indonesian pediatric COVID-19 patients.

Moreover, the mortality data of this study was presented in CFR, compared to infection fatality rate (IFR), which is a better COVID-19 mortality assessment to prevent overestimation (29). However, the usage of CFR is performed in accordance with various publications to make the comparison clearer. Many research from other countries such as the United States, Canada, United Kingdom, and European countries mainly used CFR for mortality analysis (30–33). Moreover, the data provided by the Ministry of Health over the time period of this study were in the form of CFR (6). Thus, the mortality data was presented as CFR to create an equal comparison between the data presented in this study. The calculation of IFR required comprehensive findings over positive cases in all types of patients, including the mild or atypical patients who might not test and thus omitted from the fatality rate measurement (34). Although IFR is preferable, the determination of infection as the cause of death among the subjects is difficult to achieve in retrospective data.

Finally, the study data did not include the analysis of CT value from the RT-PCR confirmed cases. The CT-value should not be solely used to reflect the status of COVID-19 disease as they can be easily misinterpreted. Some of the conditions which will affect the CT value of specimens include the methods of the swab, the viral load, and the timing of sample collection (35). In addition, no clinical autopsy was performed as it is not a routine procedure in Indonesia (36).

## Conclusion

Our study suggests a high mortality rate of children with COVID-19 in Indonesia. Thus, better public awareness for prevention and early management is needed in order to reduce the incidence and spreading of the virus.

## Data Availability Statement

The raw data supporting the conclusions of this article will be made available by the authors, without undue reservation.

## Author Contributions

AHP, NP, HS, PY, HG, RR, ADP, LN, CMS, IUg, SS, AA, HM, DS, EC, JE, LH, PO, IUt, YP, NK, AA, KK, and ABP contributed to the analysis and interpretation of the data and drafted the manuscript. AHP, NP, ABP, HS, and NK contributed to the conception and design of the manuscript. AHP, NP, HS, PY, HG, RR, ADP, LN, CS, IUg, SS, AA, HM, DS, EC, JE, LH, PO, IUt, YP, NK, AA, KK, and ABP critically revised the manuscript. All authors gave final approval and agreed to be accountable for all aspects of work ensuring integrity and accuracy.

## Funding

IPS supported all the funding for the registry.

## Conflict of Interest

The authors declare that the research was conducted in the absence of any commercial or financial relationships that could be construed as a potential conflict of interest.

## Publisher's Note

All claims expressed in this article are solely those of the authors and do not necessarily represent those of their affiliated organizations, or those of the publisher, the editors and the reviewers. Any product that may be evaluated in this article, or claim that may be made by its manufacturer, is not guaranteed or endorsed by the publisher.

## IPS COVID-19 Registry Collaborative Authors

Aris Primadi, Setia Budi Salekede, Dasril Daud, Deddy Satriya Putra, Didik Hariyanto, Didik Wijayanto, Erman, Fitri Hartanto, Herlina Dimiati, I Gusti Lanang Sidiartha, Indra Widjaja, James L. Alvin Sinaga, Munar Lubis, Mulyo Budiono, Murdoyo Rahmanoe, Mustarim, Ni Made Yuliari, Nurhandini Eka Putri, Renny Hariati, Rini Sekartini, Rocky Wilar, Sukartini, Siti Amanah Septiana, Silvia Triratna, Sjamsul Arief, Sumadiono, Taolin Fransiskus.
