# Corrigendum on: Pediatric COVID-19: Report from Indonesian pediatric society data registry

**DOI:** 10.3389/fped.2022.1024699

**Published:** 2022-10-19

**Authors:** Antonius H. Pudjiadi, Nina Dwi Putri, Hikari Ambara Sjakti, Piprim Basarah Yanuarso, Hartono Gunardi, Rosalina Dewi Roeslani, Ade Djanwardi Pasaribu, Lies Dewi Nurmalia, Catharine Mayung Sambo, I Dewa Gede Ugrasena, Santoso Soeroso, Armijn Firman, Heru Muryawan, Darmawan Budi Setyanto, Endah Citraresmi, Jaya Ariheriyanto Effendi, Lathiefatul Habibah, Prillye Deasy Octaviantie, Indriyanti Natasya Ayu Utami, Yogi Prawira, Nastiti Kaswandani, Anggraini Alam, Kurniawan Taufiq Kadafi, Aman B. Pulungan

**Affiliations:** ^1^ The Indonesia Pediatric Society, Jakarta, Indonesia; ^2^ Department of Pediatrics, Faculty of Medicine, Cipto Mangunkusumo National Central Hospital, Universitas Indonesia, Jakarta, Indonesia; ^3^ Department of Pediatrics, Faculty of Medicine, Dr. Soetomo Hospital, Universitas Airlangga, Surabaya, Indonesia; ^4^ Department of Pediatrics, Faculty of Medicine, Hasan Sadikin Hospital, Universitas Padjajaran, Bandung, Indonesia; ^5^ Department of Pediatrics, Faculty of Medicine, Dr. Kariadi Hospital, Universitas Diponegoro, Semarang, Indonesia; ^6^ Department of Pediatrics, Harapan Kita Women and Children Hospital, Jakarta, Indonesia; ^7^ Department of Pediatrics, Fatmawati Hospital, Jakarta, Indonesia; ^8^ Department of Pediatrics, Faculty of Medicine, Dr. Saiful Anwar Hospital, Universitas Brawijaya, Malang, Indonesia

**Keywords:** COVID-19, Indonesia, children, mortality, comorbidities

A corrigendum on Pediatric COVID-19: Report from Indonesian pediatric society data registry By Pudjiadi AH, Putri ND, Sjakti HA, Yanuarso PB, Gunardi H, Roeslani RD, Pasaribu AD, Nurmalia LD, Sambo CM, Ugrasena IDG, Soeroso S, Firman A, Muryawan H, Setyanto DB, Citraresmi E, Effendi JA, Habibah L, Octaviantie PD, Utami INA, Prawira Y, Kaswandani N, Alam A, Kadafi KT and Pulungan AB (2021) Front. Pediatr. 9: 716898. doi: 10.3389/fped.2021.716898

In the published article, the text in the first paragraph, **Methods** section showed an incomplete information on the intention of the original data collection and incomplete information on the information comparing the original data with Ministry of Health (MOH) COVID-19 data collection.

This sentence previously stated:

“Independent pediatricians collected and reported cases of COVID-19 on online spreadsheet created by the IPS COVID-19 task force. Data were collected from March to December 2020. These data were collected from IPS chapters COVID-19 weekly meeting and represented confirmed COVID-19 cases.”

The corrected sentence appears below:

“Independent pediatricians collected and reported cases of COVID-19 on online spreadsheet created by the IPS COVID-19 task force. The data was collected from IPS chapters COVID-19 weekly meeting and represented confirmed COVID-19 cases to support the Ministry of Health. This provision of COVID-19 pediatric data is crucial especially during the early pandemic as the official dashboard of the Ministry of Health (MoH) for the pediatric population was still in progress. The data collection for this study was conducted from March to December 2020 following the first identified COVID-19 case in Indonesia.”

This sentence previously stated:

“Data collected by total sampling include subjects’ provincial origin, age distribution, comorbidities, mortality outcome, and cause of death. For each mortality outcome, any comorbidities were recorded. This data did not represent the whole country's data as it reports individual pediatricians case findings. For a more comprehensive picture, we also report the data published by the Ministry of Health (MOH) as a comparison. These data by MOH Health account for nationwide RT-PCR-confirmed cases as reported by a certified laboratory to the MOH.”

The corrected sentence appears below:

“Data collected by total sampling include subjects’ provincial origin, age distribution, comorbidities, mortality outcome, and cause of death. For each mortality outcome, any comorbidities were recorded. This data did not represent the whole country's data as it reports individual pediatricians case findings. For a more comprehensive picture, we also report the data published by the Ministry of Health (MOH) as a comparison. These data by MOH Health account for nationwide RT-PCR-confirmed cases as reported by a certified laboratory to the MOH and were extracted from the covid19.go.id website (1). The website was supervised by the Indonesian COVID-19 Task Force which is the government official page for COVID-19 information.”

Additionally, the text in paragraph twelve, **Discussion** section showed an incomplete information on the limitation of the study.

This sentence previously stated:

“The main limitation of this study is that we could not provide comprehensive data of the suspected cases. Thus, we are unable to perform statistical analysis to measure the predictors of death in our study population. In addition, with the high number of pediatric COVID-19 cases, we predict that the number of multisystem inflammatory syndromes in children (MISC) should be higher. Previous studies in other countries such as the Latin America and UK found a prevalence of 23.2% out of 409 children and 11% in 651 children recruited. However, the authors addressed that this number could be an overestimation of the real cases due to the broad definition of MISC with no confirmatory objective parameters. Further studies are needed in order to identify the exact ratio of MISC among our population. This will be beneficial for early detection and treatment to reduce the morbidity and mortality of Indonesian pediatric COVID-19 patients.”

The corrected sentence appears below:

“The main limitation of this study is that we could not provide comprehensive data of the suspected cases. Thus, we are unable to perform statistical analysis to measure the predictors of death in our study population. The study design also did not allow the evaluation of risk or identify modifying variables for COVID-19 mortality. In addition, with the high number of pediatric COVID-19 cases, we predict that the number of multisystem inflammatory syndromes in children (MISC) should be higher. Previous studies in other countries such as the Latin America and UK found a prevalence of 23.2% out of 409 children and 11% in 651 children recruited. However, the authors addressed that this number could be an overestimation of the real cases due to the broad definition of MISC with no confirmatory objective parameters. Further studies are needed in order to identify the exact ratio of MISC among our population. This will be beneficial for early detection and treatment to reduce the morbidity and mortality of Indonesian pediatric COVID-19 patients.

Moreover, the mortality data of this study was presented in case fatality rate (CFR), compared to infection fatality rate (IFR), which is a better COVID-19 mortality assessment to prevent overestimation (2). However, the usage of CFR is performed in accordance with various publications to make the comparison clearer. Many research from other countries such as the United States, Canada, United Kingdom, and European countries mainly used CFR for mortality analysis (3–6). Additionally, the data provided by the MOH over the time period of this study were in the form of CFR (1). Thus, the mortality data was presented as CFR to create an equal comparison between the data presented in this study. The calculation of IFR required comprehensive findings over positive cases in all types of patients, including the mild or atypical patients who might not test and thus omitted from the fatality rate measurement (7). Although IFR is preferable, the determination of infection as the cause of death among the subjects is difficult to achieve in retrospective data.

Finally, the study data did not include the analysis of CT value from the RT-PCR confirmed cases. The CT-value should not be solely used to reflect the status of COVID-19 disease as they can be easily misinterpreted. Some of the conditions which will affect the CT value of specimens include the methods of the swab, the viral load, and the timing of sample collection (8). In addition, no clinical autopsy was performed as it is not a routine procedure in Indonesia (9).”

The authors state that this corrigendum does not change the scientific conclusions of the article in any way. The original article has been updated.
